# Extensive Bone Marrow Necrosis: A Rare Presentation of Acute Lymphoblastic Leukaemia

**DOI:** 10.1155/2020/8889850

**Published:** 2020-08-25

**Authors:** I. Ranathunga, N. R. Muthumala, H. W. C. K. Kulathilake, S. Weerasinghe, N. L. A. Shyamali

**Affiliations:** ^1^Professorial Medical Unit, Colombo South Teaching Hospital, Colombo, Sri Lanka; ^2^Department of Haematology, Faculty of Medicine, University of Sri Jayewardenepura, Colombo, Sri Lanka; ^3^Cancer Institute, Maharagama, Colombo, Sri Lanka; ^4^Department of Medicine, Faculty of Medicine, University of Sri Jayewardenepura, Colombo, Sri Lanka

## Abstract

**Background:**

Bone marrow necrosis (BMN) is a rare entity which presents with bone pain, fever, and peripheral cytopenia. Acute lymphoblastic leukaemia (ALL) is characterized by malignant proliferation of immature lymphocytes, and patients usually present with fatigue and bleeding manifestations. Presentation with BMN is an extremely rare finding and only few cases had been reported in the literature. *Case Presentation*. A 22-year-old male presented with nocturnal lower back ache, pleuritic central chest pain, and fever for two weeks. He was extensively investigated for a cause. His investigations revealed pancytopenia with severe neutropenia. Initial bone marrow aspiration and biopsy did not provide a positive result due to extensive necrosis. However, immunohistochemical analysis of few immature lymphoid cells on repeated BM biopsy showed evidence of acute lymphoblastic leukaemia.

**Conclusions:**

ALL usually presents with fatigue and bleeding manifestations. Presentation with BMN is extremely rare. The diagnosis was extremely challenging as this patient had only occasional atypical cells in the peripheral blood film and the repeat bone marrow (BM) biopsy showed extensive necrosis.

## 1. Background

Bone marrow necrosis (BMN) is characterized by necrosis of the medullary stroma and myeloid tissues of the haematopoietic bone marrow (BM), leaving an amorphous eosinophilic background, necrotic cells, and preserved cortical bone [1]. It is a rare disorder with only few reported cases in the literature. The reported prevalence of the disease is variable between 0.3 and 37% with low prevalence among the living [2–5]. Severity is graded as severe, moderate, and mild according to the percentage of the diameter of the biopsy showing necrosis [1]. Trephine biopsy shows disruption of BM architecture with loss of fat spaces [6]. BMN is caused by a variety of medical conditions and drugs, of which haematological malignancies play a major role. Acute lymphoblastic leukaemia (ALL) is a haematological malignancy characterized by malignant proliferation of immature lymphocytes [7]. Depending on the extent of bone marrow involvement, the clinical manifestations may range from insidious onset nonspecific symptoms to acute onset, severe, life-threatening presentations [8, 9]. Only few cases of ALL have been reported as presenting with extensive bone marrow necrosis in the literature [1].

## 2. Case Presentation

A 22-year-old previously healthy male presented with nocturnal lower back ache, pleuritic central chest pain, and intermittent fever for two weeks. He did not have other constitutional symptoms or bleeding manifestations. Prior to admission, he had been treated with antibiotics and steroids by a general practitioner. His symptoms have subsided temporarily during this treatment. On admission, he was not pale and his vital parameters were stable. He had bone tenderness over the sternum and lower lumbar spine. His cardiovascular, respiratory, abdominal, and neurological examination was normal.

On investigation, his white blood cell count (WBC) was 2.91 × 10^3^/*μ*L with 51% neutrophils, heamoglobin was 13.6 g/dL, and platelet count was 28 × 10^3^/*μ*L. Blood picture revealed bicytopenia with leucoerythroblastic picture and occasional atypical cells. Reticulocyte count was 0.8%. Initial C-reactive protein was 520 mg/L, erythrocyte sediment rate was 110 mm/1st hour, and lactate dehydrogenase (LDH) was 4834 U/L. His alkaline phosphatase was 803 U/L (30–120 U/L), gamma glutamyl transferase was 171 U/L (<55 U/L), and total bilirubin was 67 *μ*mol/L (5–21 *μ*mol/L) with increased direct fraction. His renal function and coagulation profile were normal. During the hospital stay, his WBC dropped to 0.69 × 10^3^/*μ*L with an absolute neutrophil count of 0.04 × 10^3^/*μ*L. Haemoglobin and platelets dropped to 7.4 g/dL and 6 × 10^3^/*μ*L, respectively. His viral studies including hepatitis A, hepatitis B, cytomegalovirus, Epstein-Barr, and human immunodeficiency were negative. Blood, urine, and sputum cultures were sterile. BM tuberculosis polymerase chain reaction and culture was negative.

**Figure 1:**
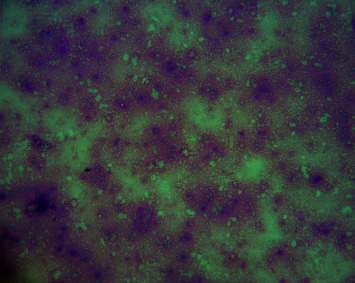
Bone marrow aspiration (Leishman stain) showing bone marrow necrosis.

**Figure 2:**
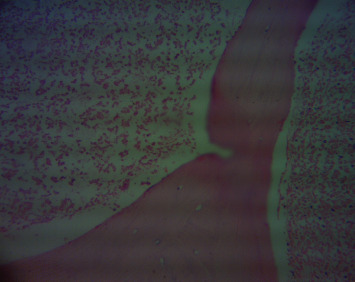
Bone marrow trephine biopsy (haematoxylin and eosin) showing extensive bone marrow necrosis.

BM aspiration and trephine biopsy revealed extensive necrosis with absence of cells. Repeated BM biopsy showed marrow spaces contained amorphous eosinophilic debris mixed with poorly demarcated cells (Figures 1 and 2). Nuclear details could not be appreciated. A clearly visible focus of immature lymphoid cells, which demonstrated nuclear terminal deoxynucleotidyl transferase (TdT) positivity on immunohistochemistry, was detected leading to the diagnosis of ALL. Immunophenotyping of peripheral blood by flow cytometry concluded 1-2% blasts of B lymphobast phenotype.

He was started on broad-spectrum antibiotics and high-dose steroids and required transfusion of several packed red cells and platelets before transfer to National Cancer Institute, Maharagama, for further management. ALL treatment protocol adapted from Hoelzer's (GMALL/BFM) 5/93 treatment regimen was started, and he has undergone the induction, consolidation 1, reinduction, and halfway of the consolidation 2 phase. He is responding well to treatment.

## 3. Discussion and Conclusions

Conditions that can cause BMN are haematological malignancies, sickle cell anaemia, infections, anorexia nervosa, haemolytic ureamic syndrome, antiphospholipid syndrome, hyperparathyroidism, and disseminated intravascular coagulation. Cytokines, antineoplastic drugs, and granulocyte colony-stimulating factors are some of the causative drugs [1]. The pathophysiology of BM necrosis can be due to several processes and mainly involves failure of the microcirculation [6]. Microcirculatory occlusion may occur as a result of fibrin thrombi formation, cytotoxic injury to vessels, tumor embolization, and tumor compression [10]. This process can be initiated by toxic effects of chemotherapy, irradiation, bacterial endotoxins, tumor cell infiltration, and cytokines [11].

Common manifestations of BMN are bone pain, fever, and peripheral cytopenia. All three cell lines may be affected while anaemia and thrombocytopenia are seen commonly [1]. Leucoerythroblastic blood picture, high LDH, and uric acid levels can be seen [1]. Our patient presented with bicytopenia and a leucoerythroblastic blood picture which can be a common presentation of hematological malignancies. The diagnosis of BMN is pathognomically made on the basis of bone marrow aspiration and biopsy findings. Due to extensive necrosis, BM aspiration is generally unsuccessful and multiple aspirations are necessary to arrive at a diagnosis [12]. Aspirated samples are opaque, whitish or reddish purple fluid. Stained smear shows amorphous pink stained material with faint outline of necrotic cells with nuclei appearing as dark stain smudges. Trephine biopsies are more informative with early stages showing pyknotic nuclei, granular cytoplasm, and indistinct cell margins. Later, nuclear karyorrhexis and complete loss of cell outlines result in amorphous eosinophilic debris with considerable loss of fat spaces [6]. Immunohistochemistry may be misleading if used to investigate neoplastic cells in necrotic areas due to loss of antigens and tendency of antibodies to adhere in a nonspecific way to necrotic tissue [6]. Though the radiological investigations may play a limited role in evaluation, MRI demonstrates a characteristic geographic pattern of signal abnormalities with diffuse and extensive involvement [13]. Characteristically, the marrow demonstrates a central region of varying signal intensity which is surrounded by a low signal intensity peripheral band [13]. Favorable prognosis depends on the underling diagnosis, especially when mild degree of bone marrow necrosis is present. BMN usually has an unfavorable outcome [9, 14]. Prolonged BMN can lead to secondary bone marrow fibrosis [1]. Median survival ranges from weeks to months [1].

ALL usually presents with fatigue and bleeding manifestations [6]. Presentation with BMN is extremely rare [1, 9]. The diagnosis was extremely challenging as this patient had only occasional atypical cells in the peripheral blood film, and the BM biopsy showed extensive necrosis. This case highlights the rare presentation of ALL and importance of repeated bone marrow examination and usage of complimentary tools in haematopathology.

## Data Availability

No data were used to support this study.

## Abbreviations

BMN: Bone marrow necrosis
ALL: Acute lymphoblastic leukaemia
BM: Bone marrow
WBC: White blood cell count
LDH: Lactate dehydrogenase
MRI: Magnetic resonance imaging.
